# Factors that influence user adherence of the Mask‐air® application

**DOI:** 10.1002/clt2.70054

**Published:** 2025-04-14

**Authors:** Anna Szylling, Boleslaw Samoliński, Filip Raciborski, Konrad Furmańczyk, Mariola Chrzanowska, Oksana Wojas, Edyta Krzych‐Fałta, Emilia Gawińska‐Drużba, Krzysztof Samoliński, Jean Bousquet, Piotr Samel‐Kowalik

**Affiliations:** ^1^ Department of Allergy and Clinical Immunology University Clinical Center of the Medical University of Warsaw Central Clinical Hospital Warszawa Mazowieckie Poland; ^2^ Department of Prevention of Environmental Hazards Medical University of Warsaw Warszawa Mazowieckie Poland; ^3^ Institute of Information Technology Warsaw University of Life Sciences Warszawa Mazowieckie Poland; ^4^ Department of Statistics and Econometrics Institute of Economics and Finance Warsaw University of Life Sciences Warszawa Mazowieckie Poland; ^5^ Department of Basic of Nursing Medical University of Warsaw Warszawa Mazowieckie Poland; ^6^ Department of Emergency Medical Services Medical University of Warsaw Warszawa Poland; ^7^ Institute of Allergology Charite Universitatsmedizin Berlin Berlin Germany; ^8^ University of Montpellier Montpellier France

**Keywords:** adherence, allergic rhinitis, CDSS, Mask‐air®

## Abstract

**Background:**

Monitoring adherence in chronic diseases remains a significant challenge. Allergic rhinitis (AR), one of the most common chronic conditions, serves as an excellent model for studying determinants of app use in monitoring adherence and health assessment during treatment. The Mask‐air® app supports clinical decision‐making by involving patients in symptom observation and promoting adherence to therapy. This study aimed to identify the defining characteristics of Mask‐air® users, describe their disease phenotype and satisfaction with the app, and explore reasons for discontinuation.

**Materials and Methods:**

Adult patients 20–44 years old suffering from AR (*n* = 198) receiving care at an allergy outpatient clinic were invited to participate in a trial using the Mask‐air® app. Investigators collected data on symptoms, administered treatments, and clinical evaluation results through questionnaires. At a follow‐up visit (*n* = 163), these were compared, and patients were questioned about their satisfaction with the app. Patients presented their app records, and those who declined or stopped using the app were asked to provide reasons in a questionnaire.

**Results:**

No distinguishing characteristics of Mask‐air® users (*n* = 131) were identified compared with those who declined the app (*n* = 67). App readiness was analyzed according to age, socioeconomic status, disease severity, comorbidities, and therapeutic modality. Respondents were categorized into: those who did not install the app (17.7%), those who installed but did not use it (16.2%), and those who installed and evaluated it (66.2%), with 15.6% failing to produce symptom monitoring records. Overall, satisfaction ratings were high though patients were critical of the app's therapeutic aspect.

**Conclusions:**

The study found no specific features distinguishing Mask‐air® users, suggesting that it can be recommended to all patients regardless of gender, socioeconomic or educational status, or disease phenotype. However, with a dropout rate of nearly 50%, it is essential for clinicians to emphasize the app's benefits to improve adherence and engagement.

## 1 INTRODUCTION

### 1.1 Background

Worldwide, rhinitis is one of the most common chronic diseases, affecting on average up to 30% of the population (median prevalence). In Europe, it may affect as much as 35% of adults and there seems to be a steady increase in its prevalence.^1^ For Poland, 40% of the country's population suffers from allergies, while allergic rhinitis (AR) is developed by 25% Poles.^2^ AR is known to impact the quality of life and sleep, but it also demonstrates a negative influence on learning and work. It constitutes a major health problem, and compared to other diseases of civilization, it significantly worsens the quality of life.^3^ The challenge in monitoring chronic diseases is how to improve patient adherence. AR, by virtue of being one of the most common chronic conditions and lifelong in its nature, is an excellent model for evaluating the factors that might determine the use of IT applications for short‐ and long‐term monitoring of adherence to therapy and patient's condition during treatment.

Mask‐air® is a global leader among similar state‐of‐the‐art applications. It was approved by OECD^4^, ^5^ and Poland's Ministry of Health as a unique solution, recommended to patients and included among healthcare benefits offered in chronic care.^6^ Mask‐air® is widespread, especially in Europe. Other than monitoring the patient's condition, it also follows pharmacotherapy in real time and allows for real‐life evaluation of drug efficacy and long‐term treatment outcomes, which is particularly important in allergen‐specific immunotherapy.

The Mask‐air® app supports clinical decision‐making processes by actively involving the patient in symptom observation and therapeutic adherence. In the context of CDSS (Clinical Decision Support System) and mHealth (mobile health), it is essential to acquire reliable data. Analyzing that data may reveal some previously unknown information about the patient and open new horizons in medical care.^7^

Accessible and reliable, the Mask‐air® app may also facilitate clinical decisions by actively involving the patient in symptom observation and adherence to therapeutic recommendations.^7^, ^8^, ^9^, ^10^, ^11^ The challenge is to encourage its use and to inspire regular users to be consistent. According to Bedard, during pollen season, app users report the presence of symptoms for 5.1 days. If they do require MP‐AzeFlu administration, it is for 10 days.^12^ Benfante, on the other hand, in her 6‐month study of patients with severe asthma receiving biological treatment, demonstrated a 51.8% adherence in app use.^13^

It is of key importance to identify the characteristics of patients (including their disease phenotype) most likely to use the Mask‐air® app for as long as possible.^14^

### 1.2 Objectives

The main objective of the study was to identify distinctive characteristics of an app user who recorded his or her daily AR symptoms in the Mask‐air® app. These characteristics included among others disease phenotype and satisfaction rating regarding app use. Additionally, reasons for refusal of study participation and rejection of app use were also subject to analysis.

## 2 MATERIAL AND METHODS

The study included adult patients (aged 20–44) receiving care in an allergy outpatient clinic. In the course of a routine visit, they were offered to participate in a trial involving the Mask‐air® app. Daily monitoring of AR symptoms and treatment was meant to support and facilitate traditional clinical patient evaluation. By means of surveys/questionnaires, investigators collected patient data regarding symptoms and administered treatment and clinical evaluation results. At the follow‐up visit, these were subsequently updated and compared. Patients allowing app use, as well as those rejecting the idea, were assessed with regard to their age, gender, socio‐economic status, education, as well as comorbidities. Subjects then evaluated the app and presented their app records, and those who had declined to install the app were requested to indicate their reasons.

### 2.1 Study design

**FIGURE 1:**
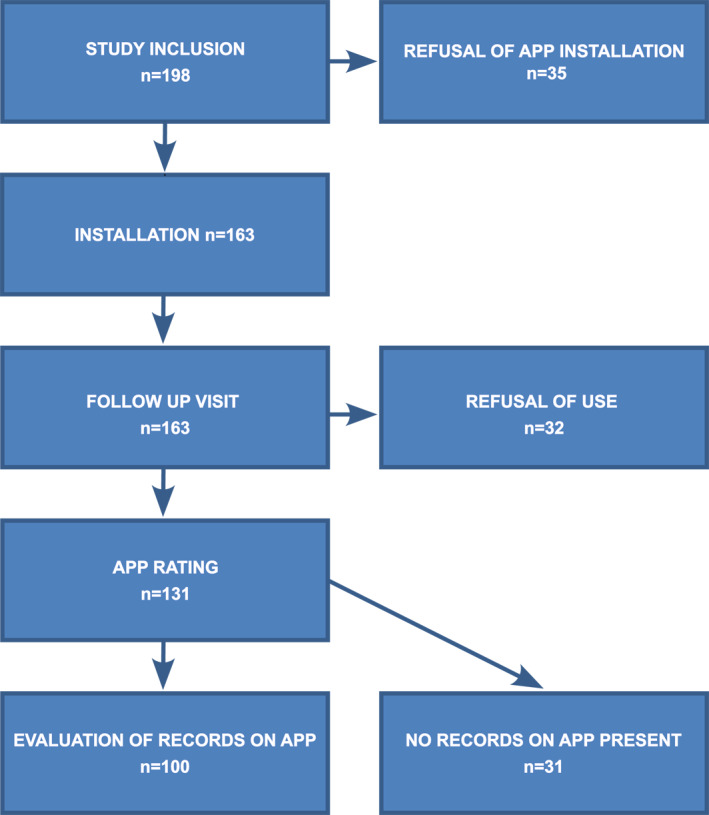
Participants of the study with Mask‐air® app.

An observational study with an interventional component: installing the Mask‐air® app on the patient's phone. Using the app, the subject could rate daily AR and/or asthma symptoms and record administration of medication (Figure 1). Patients then rated the application and—should they decline the download and use or discontinue it after previous declaration of use—they were asked to justify this decision.

### 2.2 Settings

The study was approved by the Bioethics Committee of the Medical University of Warsaw on May 13, 2019 (AKBE/213/2019). All patients participating in the study have been informed and have given their verbal consent to participate.

The study recruited AR and/or asthma patients treated at the University Clinical Center of the Medical University of Warsaw Allergy Outpatient Clinic (UCK WUM), meeting the following inclusion criteria: age from 20 to 44 and absence of other non‐allergic chronic comorbidities significantly affecting their quality of life (the studied variables were listed in the “Study Inclusion Questionnaire”).

The study design was as follows:

1. In the course of a routine visit, the patient was offered to participate in a study focusing on the Mask‐air® app. If the patient chose not to participate, the “Patient willingness to use the Mask‐air® app questionnaire” was completed. Daily monitoring of AR symptoms and treatment was intended to support traditional clinical evaluation, which is why the questionnaires included a set of questions providing for reproducibility of evaluation at the “inclusion visit” and the “follow‐up visit”: history, clinical examination, treatment evaluation and recommendations.
2. The follow‐up visit was scheduled for 2–4 weeks post study inclusion. It featured a clinical exam and responses to questions from the “follow‐up” questionnaire. Patients were also asked to rate the Mask‐air® app in the “patient satisfaction” questionnaire; after which they presented their symptoms and drug administration data recorded on the app, and discussed them with the physician. Access to data was obtained via the QRS code (www.mask‐air.org/data). If the patient failed to use the app, reasons for that change of heart were to be given in an appropriate questionnaire (“patients' willingness to use the Mask‐air® app”).
3. If in the course of the follow‐up visit the subject declared that he or she gave up on using the app, reasons for that decision were to be indicated in an appropriate questionnaire (“patients' willingness to use the Mask‐air® app”).

The MASK‐air® app is available free of charge on Google Play and the App Store. Patient data were collected anonymously. The geolocation option was selected at the patient's discretion.^15^, ^16^, ^17^

Owing to sanitary restrictions put in place in March 2020 due to the COVID‐19 pandemic, some follow‐up visits were performed remotely, via mHealth, and face to face visits were scheduled at the earliest possible date.

### 2.3 Bias

Patients were in the care of UCK WUM, which could potentially impact their status and degree of involvement in study procedures. Within the study population, subjects with university education constituted 71.7% of the sample, whereas for Poland as a whole (based on the National Census of 2021 executed by the Central Statistical Office) they made up 23.1%.

The sample was selected on the basis of previous studies^18^, ^19^ as well as comorbidity analysis.^20^

### 2.4 Study size

Patient recruitment was closed at the start of the COVID‐19 pandemic. Sample size was *n* = 198.

### 2.5 Statistical methods

Preliminary data analysis consisted of creating contingency tables and determining incidences for each feature category. A multivariate logistic regression model was then built using 18 substantively selected predictors. The response value was a binary variable (refusal to participate in the study). Based on a multivariate multiple regression model for the selected variables, the model was not found to be statistically significant (*p*‐value = 0.12 > 0.05). Single logistic regression was then performed to assess the effect of individual variables (predictors) on the probability of refusing to participate in the study. The same was done using single logistic regressions, where the explanatory variable was refusal (binary variable). Similarly, performing a chi‐square test of independence for the categorical variables (not including variables of age, time of SIT, no correlations of the refusal variable with the other categorical variables were detected [*p*‐value > 0.05]).

## 3 RESULTS

### 3.1 Participants

In the course of routine visits, 198 patients were enrolled in the study. The app was installed by 163 (82.3%), 35 (17.7%) declined to download the app and were not further monitored. During a follow‐up visit, it was determined that 32 subjects (16.2%) changed their minds and, despite previous commitment, were not using the app. 131 (66.2%) users of Mask‐air® app rated it, whereas 31 (15.6%) did not confirm any activity in terms of recording symptoms on their phones.

### 3.2 Descriptive data

Demographic characteristics of patients (Table 1).

**TABLE 1:** Patient characteristics and parameters analyzed in the population of Mask‐air® app users.

1. Age in the range of 20–44, (*n* = 198)
2. Sex: Male 118 (56.9%), female 80 (40.4%)
3. Educational status: tertiary 148 (74.7%), secondary school 26 (13.1%), secondary, but no tertiary 24 (12.1%)
4. Occupational status: employed 156 (78.8%), not employed 42 (21.2%)
5. Place of residence: Warsaw 137 (69.2%), rural area 26 (13.1%), towns of no more than 50 thousand inhabitants 20 (10.1%), cities of 50–500 thousand inhabitants 15 (7.5%)
6. Marital status: Single 113 (57.1%)/married 85 (46.9%)
7. Type of allergy: Mono‐sensitization (trees, grass, house dust mite, cat dander, molds, food allergens) versus poly‐sensitization
8. Isolated AR 100 (50.5%)
9. Seasonal AR 132 (66.7%) versus chronic AR 66 (33.3%)
10. Allergic comorbidities: a. AR and asthma 60 (30.3%) b. AR and asthma 60 (30.3%) versus degree of disease control: (Well controlled 43 patients), partially controlled or uncontrolled 17 patients c. AR and allergic conjunctivitis 40 (20.2%)
11. Therapy: a. Nonsedating OAH 142 (71.7%) b. Topical INCS 36 (18.2%), INCS + (INAH) 7 (3.5%) c. Ocular H1‐antihistamine 14 d. Allergen‐specific immunotherapy: 151 (76.2%) and its duration e. Inhaled medication: ICS or ICS + LABA 40
12. Clinical presentation of the nasal cavity: unremarkable 59 (36.2%) versus abnormal nasal patency 104 (63.8%)

Abbreviations: AR, allergic rhinitis; INCS, intranasal corticosteroids; OAH, oral H1‐antihistamine.

All subjects suffered from AR; isolated AR affected 100 patients (50.5%); the rest suffered from allergic comorbidities including 22 subjects (11.1%) showing symptoms of more than two conditions.

Concomitant asthma was found in 60 patients with AR (30.3%), of which satisfactory symptom control according to GINA^21^ was obtained in 43 subjects, 7 patients had partially controlled asthma, and in 10 patients asthma control was absent at baseline visit.

Symptoms of allergic conjunctivitis were displayed by 40 AR patients (20.2%), while symptoms of atopic dermatitis affected 21 individuals (10.6%) in the study population.

The follow‐up visit was attended by 163 patients (82.3%), with clinical improvement found in 113 of them (69%).

The majority of subjects were in the process of SITA desensitization or had already completed the treatment cycle, yet they continued therapy with oral antihistamines and INCS or Aze‐Flu.

AR treatment is described in (Table S1).

Treatment of asthma (Table S2).

Using data from patient records, patient sensitization patterns were analyzed (Table S3), and summarized by grouping together allergens causing cross‐reactivity^20^; monovalent allergy was found in 19 of 191 patients subjected to skin prick test or IgEs testing. In 7 AR patients, allergic sensitization was not confirmed and local AR was diagnosed (LAR). The study population was predominantly affected by allergies to grass pollen (156), tree pollen (143) and mugwort (111). Perennial allergen sensitization was confirmed in the following: mite in 101 subjects, cat dander in 57, and molds in 44 patients.

### 3.3 Outcome data

App users who installed and rated the app (*N* = 131), versus those who documented its use (*N* = 100).

### 3.4 Main results

**FIGURE 2:**
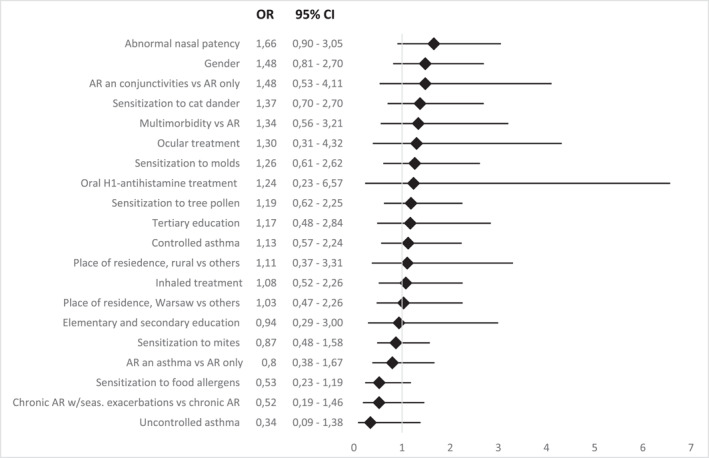
Analysis of variables in search of OR relationships for app users and those who declined to use the Mask‐air® app showed no statistical significance. However, variables showing OR > 1 were observed, suggesting a bias towards using the app, which requires further research.

We analyzed the impact of demographic factors (age, gender, occupational and economic status, type of sensitization: grasses, trees, house dust mite, cat dander and molds), allergic multimorbidity (controlled and uncontrolled asthma, allergic conjunctivitis and isolated AR), and the effect of immunotherapy and its duration. Employing a multivariate logistic regression model for the listed variables did not prove significant (*p*‐value = 0.12 > 0.05). Similarly, using single logistic regressions, where the explanatory variable was refusal (a binary variable) versus the other variables, no relationship was detected (*p*‐value > 0.05) (Figure 2).

To investigate the impact of marital status on app use, the authors used the Pearson's chi‐squared test with Yates' continuity correction that failed to reveal statistically significant differences. Additionally, app user ratios were investigated (71% of users among singles and 59% of users among non‐singles), but this evaluation lacked statistical significance owing to the limited sample size. Equality of proportions with continuity correction was utilized.

Despite the absence of statistical significance, visible trends suggest that patients allergic to grass pollen (OR: 1.89) or tree pollen (OR: 1.19) were more willing/likely to use the app, as did subjects allergic to molds (OR: 1.26) and cat dander (OR: 1.37). Patients with controlled asthma (OR: 1.13) are more likely to use the app than subjects with uncontrolled asthma (OR: 0.34), even though this result is not statistically significant. Tertiary education (OR: 1.17) and residing in rural areas (OR: 1.11) seem to slightly predispose students to app use. Study subjects with confirmed abnormalities in nasal patency (OR: 1.66) or subjected to specific immunotherapy (OR: 1.67) were more likely to use the app, despite lack of statistical significance.

**FIGURE 3:**
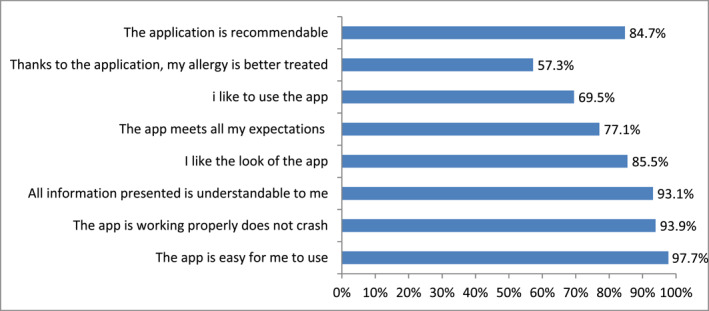
Evaluation of the MASK‐air® app by the patients (*n* = 131).

Patient ratings of the Mask‐air® app were high. It was appreciated for its simplicity of use, reliability, appearance and being understandable. Even those less willing to use the app themselves were ready to recommend it to others. The app's value in improving allergy treatment was viewed least favorably (Figure 3).

App users appreciated its utility: 103 patients had no objections or only minor ones (0–3), moderate objections (4–7) were expressed by 23 subjects, and major objections (8–10) by 5 respondents (Figure S1).

### 3.5 Other analyses

The authors analyzed the reasons offered by respondents to justify refusal of app installation and use.

Detailed data are listed in Table 2.

**TABLE 2:** Reasons for Mask‐air app installation denial and research participation refusal (*n* = 67).

Reasons for Mask‐air app installation denial	
1. Refusal of participation in the study (ONLY ONE ANSWER)	8
2. New app installation denial	12
3. After training refusal	25
4. Technical problems	22
Reasons for research participation refusal (MULTIPLE CHOICE)	
1. No mobile or it does not meet the technical requirements	6
2. No Internet on the phone	1
3. Lack of skills/reluctance to use the app on the phone	2
4. Reluctance or fear to use the new app	31
5. Technical problems with installing the app	12
6. The app is too difficult to use, despite training	0
7. Other	17

It was also confirmed that all users had access to the Internet on their smartphones. Even though the app was considered easy to use by those who did use it, as many as 22 respondents gave up on it for technical reasons (outdated model of smartphone, difficulties in app installation caused by the owner forgetting password to an e‐mail account and being unable to confirm identity during installation process, insufficient operating memory to install another app).

Twenty five patients declined to use the app after being trained in its operation. They expressed reluctance or apprehension, claimed they did not need it after all, or explained their rejection by lack of time. Twelve subjects declined to install new software on their devices.

**FIGURE 4:**
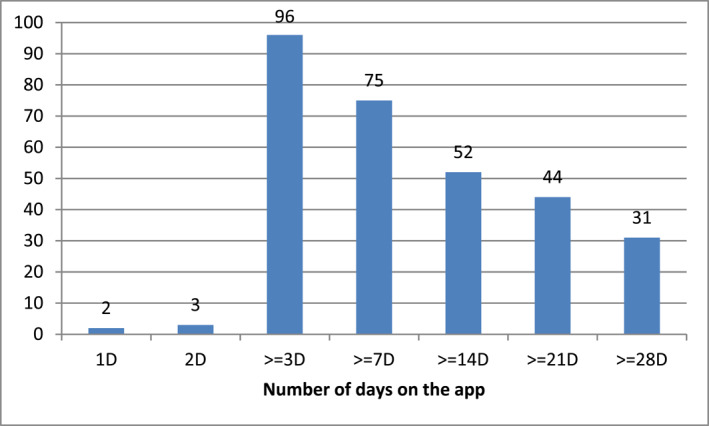
Duration of app use (*n* = 100) *Y* axis—number of users, *X* axis duration of use: 1 day, 2 days, 3 days, up to 7 days, 7–14 days, 21–28 days, 28 days and longer.

In the course of the follow‐up visit, users of Mask‐air® app presented their recorded symptoms for discussion (*n* = 100). It was found that the average time of app use in week 1 post‐installation amounted to 3 days, whereas the mean time of app use was 19 days. Subjects on AR treatment (*n* = 70) tended to be active on the app for a shorter period of time—15 days (Figure 4).

In total, app users (*n* = 100) made 1858 records of AR symptoms. Within the follow‐up period, disease control was present in 1054 records, partial control in 386, and uncontrolled AR symptoms were present in over 136 records (Table S4).

## 4 DISCUSSION

### 4.1 Key results

This research project is unique as it focuses on a validated tool already employed in the daily practice of monitoring treatment and patient well‐being, rather than remaining an experiment. Globally, Mask‐air® use was described in 61 published scientific papers. Our project is based on real life data, illustrating genuine trends in patients' readiness to use the app. Although statistical significance was not achieved (study sample may have been too small, in particular as regards the group that declined to use the app), certain tendencies were clearly illustrated. Surprisingly, the year‐round persistence of symptoms, sensitization to perennial allergens (mites, mold), and more severe course of asthma seemed to predispose users to app rejection. The underlying reasons for this tendency are unclear and require further research. What is unsurprising is that better educated patients were more eager to use the app. The difference from the less educated is, however, minor and further research may demonstrate that educational status does not play any role in the willingness to use the app. Also, unsurprising is the readiness to use the app among grass and cat allergy sufferers. Their symptoms after allergen exposure (conjunctivitis, sneezing, intense rhinorrhea) exacerbate more than is the case for perennial allergens. Similarly, why should food allergy patients use the app? All in all, it seems that multimorbidity deserves more research focus. Patients with comorbidities are an important population with more unfavorable prognoses and a more severe course of disease. In the general population of Poland, allergic multimorbidity affects 9.3%^22^ of respondents, while for the population of patients remaining in the care of specialized allergy outpatient clinic that same statistic is 49.5%, which only reveals a true profile of patients managed by specialists. Statistical significance for more pronounced app use among allergic multimorbidity patients was not confirmed, yet a comparison of the multimorbidity group with the isolated AR group may suggest that such a trend is indeed present.

What is particularly noteworthy is the strong presence of app use in SITA subjects. SITA monitoring is always long‐term and difficult to express in objective terms. Mask‐air® could be helpful in this context. It could thus be stated that app use should be mandatory in all SITA subjects, also 1 year after completing therapy.

One issue that the present project failed to explore is an attempt to find out how often and under what circumstances patients should input data into the Mask‐air® diary. Study observations seem to suggest that inputs are needed whenever the patients experience symptoms or modify treatment, but also when they are free of AR and/or asthma symptoms, but prepare to have a conversation with their attending physician about their condition. This discussion could certainly benefit from determining the specifics of disease course (seasonal vs. chronic, perennial), allergen exposure, and correlation with medications administered periodically or chronically. It seems important to remind patients regularly of the possibilities brought by mHealth and the benefits of having an app to record symptoms and medication. This issue requires further work to formulate relevant recommendations.

Why did some subjects give up on an attempt to use Mask‐air®? Patients are likely to avoid monitoring for fear of being judged; they are unwilling to contrast self‐assessment and doctor's evaluation; and they may be short of time, driven by lack of knowledge or fear of stigmatization. With monitoring apps like Mask‐air® another potential obstacle is ignorance of technology or low motivation to use technology in health assessment. According to the authors, there is one group of subjects who refuse the challenge of study enrollment altogether, and another who refuse new app installation on their device. There also appear to exist certain technical difficulties with app installation, which is surprising in this age‐group of well‐educated and professionally active patients. Consequently, it would be prudent to ensure technical support with app download and registration, possibly delivered by involved medical personnel, as was previously reported by the Kvadariene group.^23^ What was most surprising is that a major group of study subjects declined app use after having been trained in its operation. Social aspects should be taken into consideration here: “I do not want another app on my phone,” “even if I go for it, I don't want to do the clicking, it takes up too much of my time.” At the same time, all enrolled patients willingly devoted their time to filling out hardcopy surveys on their health status.

In the course of the study, the authors were able to demonstrate that even if patients do install the app, on a follow‐up visit they are likely to inform that they gave up on its use. Quoted justifications included apprehension, technical issues and other causes not specified in the questionnaire. Those reasons should be taken into account when developing new strategies for promoting app use, as they can be easily mitigated by a well‐trained medical professional.^23^ Information about the benefits offered by app use in the context of diagnosis and treatment, communicated to the patient by the doctor, may be the decisive factor in app use.^24^, ^25^ From the point of view of the physician, objective evaluation of treatment efficacy, adherence to therapy, systematic use of medication and/or its modification can be useful in supporting daily patient management.^3^, ^26^

The key to improving adherence is educating patients and increasing awareness of innovative tools for monitoring disease and treatment. These tools can help patients take better care of themselves and follow their treatment plans more consistently.^27^, ^28^ Eagerness to use an app should be viewed as a sign of patients' desire to learn more about their condition and to be more involved in treatment efficacy assessment. There is definitely room for cognitive‐behavioral education and shaping the attitude of a more aware mHealth user.

Analyzing Mask‐air® records was a part of follow‐up and as such constituted a practical use of CDSS in the process of selecting treatment modality as well as modifying therapy. App users rated its utility relatively high; they emphasized ease of use, legibility and reliability. In comparison to the cohort of Lithuanian users,^23^ ratings were 5–14 points higher. What was surprising, was an answer to the question about recommending Mask‐air® to other users (lower by 5 points, 84.7%). It may have been impacted by age group. On the other hand, our patients were more eager to rate the app (over 20%). Out of eight questions that were asked, the lowest score was given to the one assessing the app's influence on more effective allergy treatment. This might suggest that further work is needed on the app and its practical use in daily patient care. Perhaps what is needed is a change to the visualization method, allowing patients to see alteration in assessment after every daily data input.

Assessment of app utility was further supplemented by a VAS scoring. Also, here patients rated app value high, only few pointed to its limited usefulness.

Study schedule and interval between the first and follow‐up visits of app use. On average it amounted to 19 days, while—surprisingly—for patients on AR therapy it did not exceed 15 days. Pfaar confirmed that in pollination season the mean time of app use was 17.5 days.^29^

### 4.2 Limitations

Single‐center study.

The COVID‐19 pandemic influenced study execution. Recruitment of new patients had to be discontinued, and follow‐ups took the form of remote visits, with face‐to‐face contact being scheduled at the earliest possible date.

No patients with isolated asthma were enrolled in the study despite it being assumed by inclusion criteria. In a German MAS study by Gought, it was found that asthma was more prevalent than a comorbidity rather than an isolated disease entity.^30^

Patients were not asked to reveal the total time they spent using their smartphones or using health‐related apps. One of the reasons for app refusal, marked as “Other,” could potentially be associated with a high number of various apps already installed on mobile devices and blocking their operating memory. Not all smartphones allow for downloading additional software.

The age group of 20–44 was selected in order to include patients actively searching for solutions to their health problems online, also owing to dissatisfaction with current treatment of their complex and severe allergic multimorbidity. For the same reason, younger patients were intentionally disregarded. The older age group was not included for fear of digital exclusion.

### 4.3 Interpretation

Study subjects, being in the care of allergy outpatient clinic, are a selected group of patients with the most severe symptoms of AR. Allergic multimorbidity affects them five times more often than the general population.^22^ Mortz demonstrated that patients with allergies at the age of 14 presented with a severe course of AR at age 29.^31^ Allergic multimorbidity, chronic nature of symptoms and being accustomed to their presence, as well as long‐term pharmacotherapy may all discourage patients of this age group from searching for new mHealth solutions.^7^ However, an experienced team of medical professionals and identification of barriers play an important role to play in ensuring effective use of technology. It is also important to repeatedly remind patients that data is collected anonymously, and to underline the benefits specific patients can enjoy in their own environment.

It is definitely advisable to recommend app use to every patient. This was confirmed by high ratings of satisfaction issued by app users. To the physician, information from app records is an invaluable support in evaluating symptom severity or deciding to modify treatment and assessing its efficacy.

There exists a need for cognitive‐behavioral education and shaping the attitude of an aware mHealth user.

### 4.4 Generalizability

We are now witnessing a breakthrough in the practical utilization of mHealth, personalized technology‐based solutions facilitating patient care in all fields of medicine. Following a multitude of apps and the potential they offer may prove confusing for the user, which is why proper app selection is essential. Apps need to be further developed and their functionalities enhanced to provide more options and to be more attractive to the user. Patients will then be encouraged to use digital tools to find new health‐related information on it, such as air pollution, current weather conditions, or the presence of pollen in their immediate surroundings.

Identifying factors that can lead to app rejection creates an opportunity to adapt and offer proper support reflecting patients' needs.

Considering the high financial burden generated by modern medical treatment, electronic devices are likely to play an ever more important role in the process of diagnosis, treatment and health education. They also contribute to new options in providing care to patients.

## 5 CONCLUSIONS

No distinguishing characteristics of Mask‐air® users were identified, indicating that the Mask‐air® app can be recommended to all patients, regardless of gender, socioeconomic and educational background, or disease phenotype. However, given the dropout rate of nearly half of the patients, it is crucial for doctors to emphasize the benefits of using the app to ensure better adherence and engagement.

## CONFLICT OF INTEREST STATEMENT

The authors declare no conflicts of interest.

## Supporting information

Supporting Information S1

## Data Availability

Data available on request due to privacy/ethical restrictions.

## References

[1] Savouré M , Bousquet J , Jaakkola JJK , Jaakkola MS , Jacquemin B , Nadif R . Worldwide prevalence of rhinitis in adults: a review of definitions and temporal evolution. Clin Transl Allergy. 2022;12(3):e12130. 10.1002/clt2.12130 35344304 PMC8967272

[2] Samolinski B , Sybilski AJ , Raciborski F , et al. Prevalence of rhinitis in Polish population according to the ECAP (Epidemiology of Allergic Disorders in Poland) study. Otolaryngol Pol. 2009;63(4):324‐330. 19999749 10.1016/s0030-6657(09)70135-0

[3] Bousquet J , Schunemann HJ , Samolinski B , et al. Allergic Rhinitis and its Impact on Asthma (ARIA): achievements in 10 years and future needs. J Allergy Clin Immunol. 2012;130(5):1049‐1062. 10.1016/j.jaci.2012.07.053 23040884

[4] Council Directive 93/42/EEC of 14 June 1993 concerning medical devices. In: The TCO, Communities E , eds. Official Journal L 169; 1993:0001‐0043.

[5] Directive 95/46/EC of the European Parliament and of the Council of 24 October 1995 on the protection of individuals with regard to the processing of personal data and on the free movement of such data. Official Journal L 281; 1995:0031‐0050.

[6] Aplikacje Certyfikowane MZ. które znajdują się w Portfelu Aplikacji Zdrowotnych (PAZ). https://www.gov.pl/web/zdrowie/aplikacje‐certyfikowane‐mz‐ktore‐znajduja‐sie‐w‐portfelu‐aplikacji‐zdrowotnych‐paz

[7] Szylling A , Raciborski F , Wojas O , et al. Why the role of mHealth in allergy diagnosis and treatment adherence cannot be overlooked. Clin Transl Allergy. 2023;13(10):e12298. 10.1002/clt2.12298 37876036 PMC10580813

[8] Sousa‐Pinto B , Sá‐Sousa A , Vieira RJ , et al. Behavioural patterns in allergic rhinitis medication in Europe: a study using MASK‐air(®) real‐world data. Allergy. 2022. 10.1111/all.15275 35258105

[9] Bousquet J , Anto JM , Bachert C , et al. ARIA digital anamorphosis: digital transformation of health and care in airway diseases from research to practice. Allergy. 2021;76(1):168‐190. 10.1111/all.14422 32512619

[10] Bousquet J , Ansotegui IJ , Anto JM , et al. Mobile technology in allergic rhinitis: evolution in management or revolution in health and care? J Allergy Clin Immunol Pract. 2019;7(8):2511‐2523. 10.1016/j.jaip.2019.07.044 31445223

[11] Bousquet J , Schunemann HJ , Fonseca J , et al. MACVIA‐ARIA Sentinel NetworK for allergic rhinitis (MASK‐rhinitis): the new generation guideline implementation. Allergy. 2015;70(11):1372‐1392. 10.1111/all.12686 26148220

[12] Bédard A , Basagaña X , Anto JM , et al. Treatment of allergic rhinitis during and outside the pollen season using mobile technology. A MASK study. Clin Transl Allergy. 2020;10(1):62. 10.1186/s13601-020-00342-x 33298191 PMC7726888

[13] Benfante A , Sousa‐Pinto B , Pillitteri G , et al. Applicability of the MASK‐air(®) App to severe asthma treated with biologic molecules: a pilot study. Int J Mol Sci. 2022;23(19):11470. 10.3390/ijms231911470 36232771 PMC9569460

[14] Sousa‐Pinto B , Anto A , Berger M , et al. Real‐world data using mHealth apps in rhinitis, rhinosinusitis and their multimorbidities. Clin Transl Allergy. 2022;12(11):e12208. 10.1002/clt2.12208 36434742 PMC9673175

[15] Olsen J , Bréart G , Feskens E , et al. Directive of the European Parliament and of the council on the protection of individuals with regard to the processing of personal data and on the free movement of such data. The International Epidemiological Association‐IEA European Epidemiological Group. Int J Epidemiol. 1995;24(2):462‐463. 10.1093/ije/24.2.462 7635612

[16] Samreth D , Arnavielhe S , Ingenrieth F , et al. Geolocation with respect to personal privacy for the Allergy Diary app – a MASK study. World Allergy Organ J. 2018;11(1):15. 10.1186/s40413-018-0194-3 30061979 PMC6048852

[17] Bousquet J , Caimmi DP , Bedbrook A , et al. Pilot study of mobile phone technology in allergic rhinitis in European countries: the MASK‐rhinitis study. Allergy. 2017;72(6):857‐865. 10.1111/all.13125 28072463

[18] Burney PG , Luczynska C , Chinn S , Jarvis D . The European community respiratory health survey. Eur Respir J. 1994;7(5):954‐960. 10.1183/09031936.94.07050954 8050554

[19] Samoliński B . Epidemiologia Chorób Alergicznych w Polsce (ECAP). Pol J Allergol. 2014;1(1):1018. 10.1016/j.alergo.2014.03.008

[20] Raciborski F , Bousquet J , Namyslowski A , et al. Correction to: dissociating polysensitization and multimorbidity in children and adults from a Polish general population cohort. Clin Transl Allergy. 2019;9:23. 10.1186/s13601-019-0263-x 30988897 PMC6449926

[21] 2019 GINA report, global strategy for asthma management and prevention . https://ginasthma.org/reports/2019

[22] Raciborski F , Bousquet J , Bousqet J , et al. Dissociating polysensitization and multimorbidity in children and adults from a Polish general population cohort. Clin Transl Allergy. 2019;9(1):4. 10.1186/s13601-019-0246-y 30792849 PMC6369558

[23] Kvedarienė V , Biliute G , Didziokaitė G , et al. Mobile health app for monitoring allergic rhinitis and asthma in real life in Lithuanian MASK‐air users. Clin Transl Allergy. 2022;12(9):e12192. 10.1002/clt2.12192 36178186 PMC9510653

[24] Lombardi C , Bonini M , Passalacqua G . The role of mobile apps in allergic respiratory diseases: an Italian multicentre survey report. Eur Ann Allergy Clin Immunol. 2018;50(6):268‐272. 10.23822/EurAnnACI.1764-1489.76 30318869

[25] Glattacker M , Boeker M , Anger R , et al. Evaluation of a mobile phone app for patients with pollen‐related allergic rhinitis: prospective longitudinal field study. JMIR Mhealth Uhealth. 2020;8(4):e15514. 10.2196/15514 32301735 PMC7195669

[26] Gálffy G , Emmeluth M , Koltun A , Kopietz F , Nguyen DT , Kuhl HC . Allergic rhinitis therapy decisions during a routine consultation: a multicenter, cross‐sectional survey. J Asthma Allergy. 2021;14:335‐345. 10.2147/jaa.s291747 33854340 PMC8039052

[27] Boecking B , Stoettner E , Brueggemann P , Mazurek B . Emotional self‐states and coping responses in patients with chronic tinnitus: a schema mode model approach. Front Psychiatr. 2024;15:1257299. 10.3389/fpsyt.2024.1257299 PMC10916791 38449502

[28] Gómez‐Redondo P , Valenzuela PL , Martínez‐de‐Quel Ó , et al. The role of supervision and motivation during exercise on physical and mental health in older adults: a study protocol for a randomized controlled trial (PRO‐Training project). BMC Geriatr. 2024;24(1):274. 10.1186/s12877-024-04868-8 38509514 PMC10953175

[29] Pfaar O , Sousa‐Pinto B , Devillier P , et al. Effects of allergen immunotherapy in the MASK‐air study: a proof‐of‐concept analysis. Allergy. 2021;76(10):3212‐3214. 10.1111/all.14955 34028052

[30] Gough H , Grabenhenrich L , Reich A , et al. Allergic multimorbidity of asthma, rhinitis and eczema over 20 years in the German birth cohort MAS. Pediatr Allergy Immunol. 2015;26(5):431‐437. 10.1111/pai.12410 26011739 PMC4744942

[31] Mortz CG , Andersen KE , Poulsen LK , Kjaer HF , Broesby‐Olsen S , Bindslev‐Jensen C . Atopic diseases and type I sensitization from adolescence to adulthood in an unselected population (TOACS) with focus on predictors for allergic rhinitis. Allergy. 2019;74(2):308‐317. 10.1111/all.13630 30307618

