# Supplementary material for: Factors that influence user adherence of the Mask‐air® application
Source: Clin Transl Allergy. 2025 Apr 14;15(4):e70054. doi: 10.1002/clt2.70054 (PMC11996621; doi:10.1002/clt2.70054)
Supplement: Supplementary file 1 — Supporting Information S1 [file CLT2-15-e70054-s001.docx]

Table S1 Current treatment of allergic rhinitis declared by the patient, inclusion in the study, first visit combination of an INCS and an INAH (combination of intranasal fluticasone propionate and azelastine hydrochloride)

|  |  | **First visit** | **Follow-up visit** |
| --- | --- | --- | --- |
|  | Nonsedating oral H1-antihistamine (OAH) | 142 (71.7%) | 118 |
|  | Ocular H1-antihistamine | 14 (7.1) | 5 |
|  | Intranasal corticosteroid ((INCS) | 36 (18.2%) | 33 |
|  | Combination of an ( INCS) and an (INAH) | 7 (3.5 %) | 13 |
|  | Specific allergen immunotherapy | 151 (76.2%) |  |
|  | n | 198 (100%) | 163 (82.3%) |

Table S2. List of asthma controller treatment during/ on the day the First visit and Follow-up visit

|  | **First visit** | **Follow-up visit** |
| --- | --- | --- |
| Controller treatment today | 40 | 35 |
| Inhaled corticosteroid (ICS) in the last week | 13 | 12 |
| Inhaled corticosteroid (ICS)+long- acting β2-agonist  (LABA) in the last week | 31 | 28 |
| Reliever: short- acting β2-agonist (SABA) in the last week | 3 | 1 |
| Daily leukotriene receptor antagonist (LTRA)in the last week | 11 | 9 |

Table S3. Skin prick test results (Allergopharma preparations), positive ≥3mm

| **Confirmed sensitization to allergen** | **Number of sensitized patients** |
| --- | --- |
| 1. **Group of Trees:**   Birch  Alder  Hazel  Trees mono  Trees 2x  Trees 3x | **143**  142  124  117  14  18  111 |
| 1. **Group of grasses:**   Grasses  Rye  Grasses mono  Grasses and rye | **156**  154  142  16  140 |
| 1. **Mugwort** | **111** |
| 1. **Molds:** | **44** |
| 1. **Mite** | **101** |
| 1. **Cat dander** | **57** |
| 1. **Selected food allergens** | **28** |
| 1. **Summary of sensitization to allergen groups**:   1 group, mono-sensitization  2x  3x  4x  5x  6x | **191**  19  40  48  58  19  7 |

Table S4. User activity of the Mask-air app (n=131)

| Use of the Mask-air app | N=131 |
| --- | --- |
| Registration during the first visit, app evaluation  Those who presented the records  Those who did not present the results for selected reasons: (deleted the app, changed their phone, lost their phone, forgot their phone) | 131  100  31 |
| Confirmed users of the app n=100  Total number of records  Time of data collection from 2019-05-10 to 2020-10-31 | 1858 |
| Days of symptom control  Days of partial control  Days of no symptom control | 89  69  39 |
| Number of records when symptom control  Number of records when partial control  Number of records when no symptom control | 1054  386  136 |
| Number of patients treated/ records | 70/1061 |
| Mean time of app use, days | 15 |
| Average days of use for the first week | 3 |

Figure S1. Assessment of app utility for users (n=131) expressed as a VAS score.

Y axis –number of app users, X axis – VAS score, where: „0” – the most useful, „10” – the least useful.
